# NKG2D^+^CD4^+^ T Cells Kill Regulatory T Cells in a NKG2D-NKG2D Ligand- Dependent Manner in Systemic Lupus Erythematosus

**DOI:** 10.1038/s41598-017-01379-y

**Published:** 2017-04-28

**Authors:** Di Yang, Zhiqiang Tian, Mengjie Zhang, Weibing Yang, Jun Tang, Yuzhang Wu, Bing Ni

**Affiliations:** 1 Institute of Immunology, PLA, Third Military Medical University, Chongqing, 400038 P.R. China; 2 Department of Pathophysiology and High Altitude Pathology/Key Laboratory of High Altitude Environment Medicine (Third Military Medical University), Ministry of Education/Key Laboratory of High Altitude Medicine, College of High Altitude Military Medicine, Third Military Medical University, Chongqing, 400038 P.R. China; 3 Department of Dermatology, 181st Hospital of PLA, Guilin, 541002 P.R. China; 4 Department of Dermatology, 105th Hospital of PLA, Hefei, 230001 P.R. China

## Abstract

Systemic lupus erythematosus (SLE) features a decreased pool of CD4^+^CD25^+^Foxp3^+^ T regulatory (Treg) cells. We had previously observed NKG2D^+^CD4^+^ T cell expansion in contrast to a decreased pool of Treg cells in SLE patients, but whether NKG2D^+^CD4^+^ T cells contribute to the decreased Treg cells remains unclear. In the present study, we found that the NKG2D^+^CD4^+^ T cells efficiently killed NKG2D ligand (NKG2DL)^+^ Treg cells *in vitro*, whereby the surviving Treg cells in SLE patients showed no detectable expression of NKG2DLs. It was further found that MRL/lpr lupus mice have significantly increased percentage of NKG2D^+^CD4^+^ T cells and obvious decreased percentage of Treg cells, as compared with wild-type mice. Adoptively transferred NKG2DL^+^ Treg cells were found to be efficiently killed in MRL/lpr lupus mice, with NKG2D neutralization remarkably attenuating this killing. Anti-NKG2D or anti-interferon-alpha receptor (IFNAR) antibodies treatment in MRL/lpr mice restored Treg cells numbers and markedly ameliorated the lupus disease. These results suggest that NKG2D^+^CD4^+^ T cells are involved in the pathogenesis of SLE by killing Treg cells in a NKG2D-NKG2DL-dependent manner. Targeting the NKG2D-NKG2DL interaction might be a potential therapeutic strategy by which Treg cells can be protected from cytolysis in SLE patients.

## Introduction

Systemic lupus erythematosus (SLE) is a chronic autoimmune disease (AID) characterized by the destruction of immune regulation^1^, in which defects in Treg cell numbers and/or function have been observed both in patients and in a lupus-prone mouse model^2–6^. The mechanisms underlying the defects or deficiency of T regulatory (Treg) cells are variable, and include reduced capacity of suppression function, increased Treg cell apoptosis and the inhibition of Treg cell differentiation in the presence of high serum levels of proinflammatory cytokines such as interferon (IFN)-α, IFN-γ, interleukin (IL)-6, IL-1β and IL-17^7–9^. Most previous studies have focused on how Treg cells regulate effector T cells, B cells, natural killer (NK) cells or antigen-presenting cells (APCs)^10–12^. However, whether and how T cells regulate Treg cells in SLE is unclear.

NKG2D^+^CD4^+^ T cells are a novel subset of T lymphocytes reported in many AIDs, such as rheumatoid arthritis, Crohn’s disease and granulomatosis with polyangiitis (GPA) (Wegener’s); these cells interact with NKG2D ligand (NKG2DL)-positive cells, such as synoviocytes, endothelial and epithelial cells. thereby causing autoreactive T cell stimulation and AID^13–15^. TNF-α and IL-15, which are abundant in inflamed local tissue or peripheral blood, can induce NKG2D expression on CD4^+^ T cells in RA, GPA and SLE^13, 14, 16, 17^. Recently, NKG2D^+^CD4^+^ T cells have been reported to produce IL-17 in type 2 diabetes (T2D) and to exert cytotoxic effects in Crohn’s disease and GPA^14, 17, 18^.

The NKG2DLs are stress-induced proteins, including the major histocompatibility complex (MHC) class I-chain-related (MIC)A, MICB and UL16-binding protein (ULBP) in humans, as well as the UL16-binding protein-like transcript 1 (Mult-1), histocompatibility 60 (H60) and retinoic acid early inducible-1 (Rae-1) in mice^19^. Normally, these ligands have a restricted tissue distribution in intestinal epithelium and can be induced by stresses such as viral and bacterial infections, malignant transformation and proliferation, thereafter acting as danger signals to alert NK cells and CD8^+^ T lymphocytes by engaging the NKG2D activating receptor^20–22^. Therefore, APCs, endothelial and epithelial cells express high levels of NKG2DLs, such as MICA/B as in AID, and interact with NKG2D^+^ cells to aggravate inflammation^21^. Indeed, increased serum level of soluble (s)MICA and extensive expression of MICA in kidneys of patients with lupus nephritis have been implicated in lupus pathogenesis^23, 24^, and expression of the other NKG2DL, ULBP, has been detected in hair follicle dermal sheath in alopecia areata^25, 26^; however, whether NKG2DLs are expressed on T cells in SLE is not known.

Given the aforementioned reduction in Treg cell numbers and abnormal expansion of NKG2D^+^CD4^+^ T cells, we hypothesized that these two cell subsets may be correlated. Furthermore, because we previously observed aberrant NKG2D expression on CD4^+^ T cells in SLE^16^, we hypothesized that the NKG2D-NKG2DL interaction links NKG2D^+^CD4^+^ T cells and Treg cells in this disease. Here, we provide evidence of significant upregulation of ULBP (Mult-1 in mice) on Treg cells upon lupus serum stimulation. Antibody (Ab) neutralization and direct cytokine stimulation experiments further verified that IFN-α is the main factor in SLE serum that induces ULBP and Mult-1 expression on the Treg cells. NKG2DL^+^ Treg cells were killed by NKG2D^+^CD4^+^ T cells, the latter of which secreted granzyme and perforin to induce apoptosis of the target cells. However, blocking NKG2D-NKG2DL binding markedly inhibited this cytotoxicity *in vitro*. Moreover, adoptive transfer experiments suggested that NKG2DL^+^ Treg cells could be killed in a lupus-prone mouse model (MRL/lpr mice), which could be protected by NKG2D blocking. Therefore, our results indicate that NKG2D^+^CD4^+^ T cells can kill Treg cells induced to express NKG2DL by IFN-α in a NKG2D-NKG2DL-dependent manner, thereby leading to the dramatic reduction in Treg numbers in SLE and highlighting the NKG2D-NKG2DL interaction as a novel therapeutic target for SLE treatment.

## Materials and Methods

### Mice

Female C57BL/6 mice (referred to here as B6 mice), B6.MRL-Fas^lpr^/J mice (referred to here as B6.MRL/lpr mice) and NZBW/F1 mice (The Jackson Laboratory, Bar Harbor, ME, USA) were maintained in the Animal Facility under pathogen-free conditions according to the guidelines for experimental animals at the Third Military Medical University, Chongqing, China. All animal studies were approved by the Ethics Committee for Animal Experimentation of the Third Military Medical University.

### Subjects and blood samples

Peripheral blood samples were obtained from 66 untreated patients with SLE from the Southwest Hospital (Chongqing, China). They all met the American College of Rheumatology (ACR) criteria for SLE^27^. Disease activity was scored according to the SLE Disease Activity Index (SLEDAI) scoring system, with mild/moderate having score 5 < SLEDAI < 14 and severe having score >14. The detailed clinical information of subjects is listed in Supplementary Table 1. Patients with Sjogren syndrome (n = 5), systemic scleroderma (n = 8) and rheumatoid arthritis (n = 8), were used as disease controls. Healthy volunteer controls (HC; n = 46) with no family history of AID were recruited from Center of Health Examination of the Southwest Hospital. All subjects enrolled in the study provided written informed consent, and all activities were approved by the Ethics Committee of the Third Military Medical University, Chongqing, China. The methods in this study were carried out in accordance with the approved guidelines.

### Antibodies and flow cytometry (FCM)

The following anti-human and mouse monoclonal antibodies (eBioscience, San Diego, CA, USA) were used for multiparameter FCM, respectively, anti-CD3-FITC (OKT3 and 17A2), anti-CD8-PerCPCy5.5 (SK1 and 53-6.7), anti-CD14-FITC (61D3 and Sa2-8), anti-CD19-PECy7 (HIB19 and Sa2-8), anti-CD4- PerCPCy5.5 (OKT-4 and GK1.5), anti-APC-CD25 (BC96 and PC61.5) and anti-PE-NKG2D (1D11 and CX5). The anti-human antibodies of anti-CD56-PE (CMSSB), anti-TIM3-APC (F38-2E2), anti-CTLA-4-FITC(14D3), anti-PD1-APC (eBioJ105 (J105)), anti-CD45RA-PerCPCy5.5 (HI100), anti-CD45RO-APC (UCHL1), anti-MICA/B- Alexa Fluor 488 (6D4), and anti-perforin-FITC (dG9), anti-FasL-PE (NOK-1), anti-granzyme B-FITC (496B), anti-IFN-γ-FITC (GZ-4), anti-IL17A-PerCPCy5.5 (eBio64CAP17), anti-IL-4-PECy7 (8D4-8), anti-CD27-APC (LG.3A10), anti-CCR7-APC (3D12), anti-HLA-DR-APC (LN3), anti-CD38-APC (HIT2) and anti-FoxP3-APC (236 A/E7), and the Fixable Viability Dye and Annexin V Apoptosis Detection Kit were all obtained from eBioscience. The anti-ULBP1-FITC (MAB 170818), anti-ULBP2-FITC (MAB 165903), anti-ULBP3-FITC (MAB 166510), anti-H60-FITC (MAB 205326), anti-Mult-1-PE (MAB 237104) and anti-Rae-1-APC (MAB 186107) were all from R&D Systems (Minneapolis, MN, USA). The anti-human INF-α receptor (IFNAR)1 (MMHAR-1) and IFNAR2 (MMHAR-2) conjugated with PE were obtained from PBL Assay Science (Piscataway, NJ, USA). The anti-mouse IFNAR1-PE (MAR1-5A3) was obtained from eBioscience, and the polyconal antibody anti-mouse IFNAR2-PE was from R&D Systems. Stained cells were analyzed with a FACS Canto instrument and data were analyzed with FlowJo software (Treestar, Ashland, OR, USA).

### Isolation purified cell, stimulation and cytolysis assays

Purified Treg cells, CD14^+^ monocytes and NKG2D^+^CD4^+^ T cells were obtained from peripheral blood mononuclear cells (PBMCs) from the HCs and SLE patients, respectively, using magnetic beads (Stem cell Technologies, Vancouver, BC, Canada) or via cell sorting (FACSAria; BD Biosciences, San Jose, CA, USA). The purity for each population was above 95%. The sorted Treg cells were cultured in RPMI 1640 supplemented with 10% FBS in the presence of HC serum or lupus serum with or without anti-INFAR2 blocking, or stimulated by human recombinant IFN-α (1000 U/mL) or PBS control; then, viable Treg cells were sorted (FACSAria) and subjected to FCM for detection of ULBP expression. In addition, viable Treg cells stimulated by SLE serum or IFN-α were sorted (FACSAria), labeled with ^51^Cr and irradiated (25 Gy), co-cultured for 6–8 h with NKG2D^+^CD4^+^ T cells from SLE patients at different effector:target ratios, and subjected to standard ^51^Cr-release assays^28^. In Abs blocking experiments, neutralizing anti-NKG2D mAb (5 μg/mL; eBioscience), anti-ULBP1-3 mAb (5 μg/mL; R&D Systems), and anti-FasL mAb (5 μg/mL; eBioscience) were added to the co-culture system.

### Adoptive transfer of serum-stimulated Treg cells to lupus or control mice

Purified Treg cells were sorted from splenocytes freshly isolated from C57BL/6 control mice by magnetic beads sorting (CD4^+^CD25^+^ Regulatory T Cell Isolation Kit, Miltenyi, Bergisch Gladbach, Germany). The sorted Treg cells were then stimulated with B6 control mice serum or B6.MRL/lpr mice serum in the absence or presence of murine recombinant IFN-α (1000 U/mL; PeproTech, Rocky Hill, NJ, USA) for 18–24 h. In neutralization experiments, anti-IL-1R, anti-IL-6R, anti-TGF-β, anti-TNF-α, anti-IFNγR and anti-IFNαR were added to the culture system, respectively. All these Tregs were analyzed for NKG2DL Mult-1 expression by FCM. In addition, 2 × 10^6^ viable serum-induced Treg cells labeled with CFSE were transferred into 16-week-old female B6.MRL/lpr mice or age and sex-matched control C57BL/6 mice by injection in the tail vain. Anti-mouse NKG2D mAb (CX5; eBioscience) or control IgG (cIg) (200 μg/injection) was injected 8 h before transfer experiments. The recipient mice were then sacrificed and CFSE^+^ Treg cell frequency was analyzed in gated CD4^+^ T cells of peripheral blood.

### Kidney histology and immunofluorescence staining

OCT-embedded kidney tissue sections from MRL/lpr mice were stained with hematoxylin and eosin (H&E) or antibodies against mouse C3, IgG and IgM followed by the fluorescence-labeled secondary antibodies, all of which were from Abcam (Cambridge, MA, USA). Glomerular, tubulointerstitial and vascular damage were evaluated on H&E-stained slides using a semiquantitative scoring system (0, 1, 2 and 3)^29^. Fluorescence staining of slides were scored (0–3) in a masked fashion by an experienced renal pathologist for the intensity and coverage of immunofluorescence in the glomerulus (0, no staining; 1+, mild staining; 2+, moderate staining; 3+, strong staining).

### Treatment with anti–IFNAR Ab and anti-NKG2D Ab

Female B6.MRL/lpr mice were treated with anti-IFNAR1 mAb (MAR1-5A3; eBioscience) or anti-NKG2D mAb (CX5; eBioscience) as previously described^30, 31^. Anti-IFNAR1 mAb or cIg injections were started at 15 wk of age, when the appearance of disease manifests, as suggested by detectable autoantibody titers and proteinuria (i.p., 500 μg for three consecutive days, followed by 250 μg three times per week until experiment termination). Similarly, injections of anti-NKG2D mAb or cIg were started at the same age (i.p. on days 1 and 5 with 8 μg/g of anti-NKG2D mAb (CX5) twice weekly).

### Measurement of cytokine level

Supernatants from serum of SLE patients and MRL/lpr mice were stored at −80 °C until use. The levels of serum and supernatant IFN-α, TNF-α, IL-1β, IL-6, TGF-β and IFN-γ were then measured using an ELISA kit (R&D Systems). Supernatants of the co-cultures comprised of NKG2D^+^CD4^+^ T cells and serum-induced Treg cells were collected for measurement of perforin and granzyme B by ELISA kits (LifeSpan BioSciences, Seattle, WA, USA and Genway Biotech, San Diego, CA, USA, respectively).

### Statistical analysis

Statistical analysis was performed using GraphPad Prism 5 software. The Mann-Whitney test was used for comparisons between patients and controls. Comparisons between different groups were performed with Student’s t-tests or one-way ANOVA with the Tukey’s multiple comparison test, and Spearman’s test was used for correlation studies. P < 0.05 was considered significant.

## Results

### Aberrant expansion of cytotoxic NKG2D^+^CD4^+^ T cells in the peripheral blood in patients with SLE

**Figure 1:**
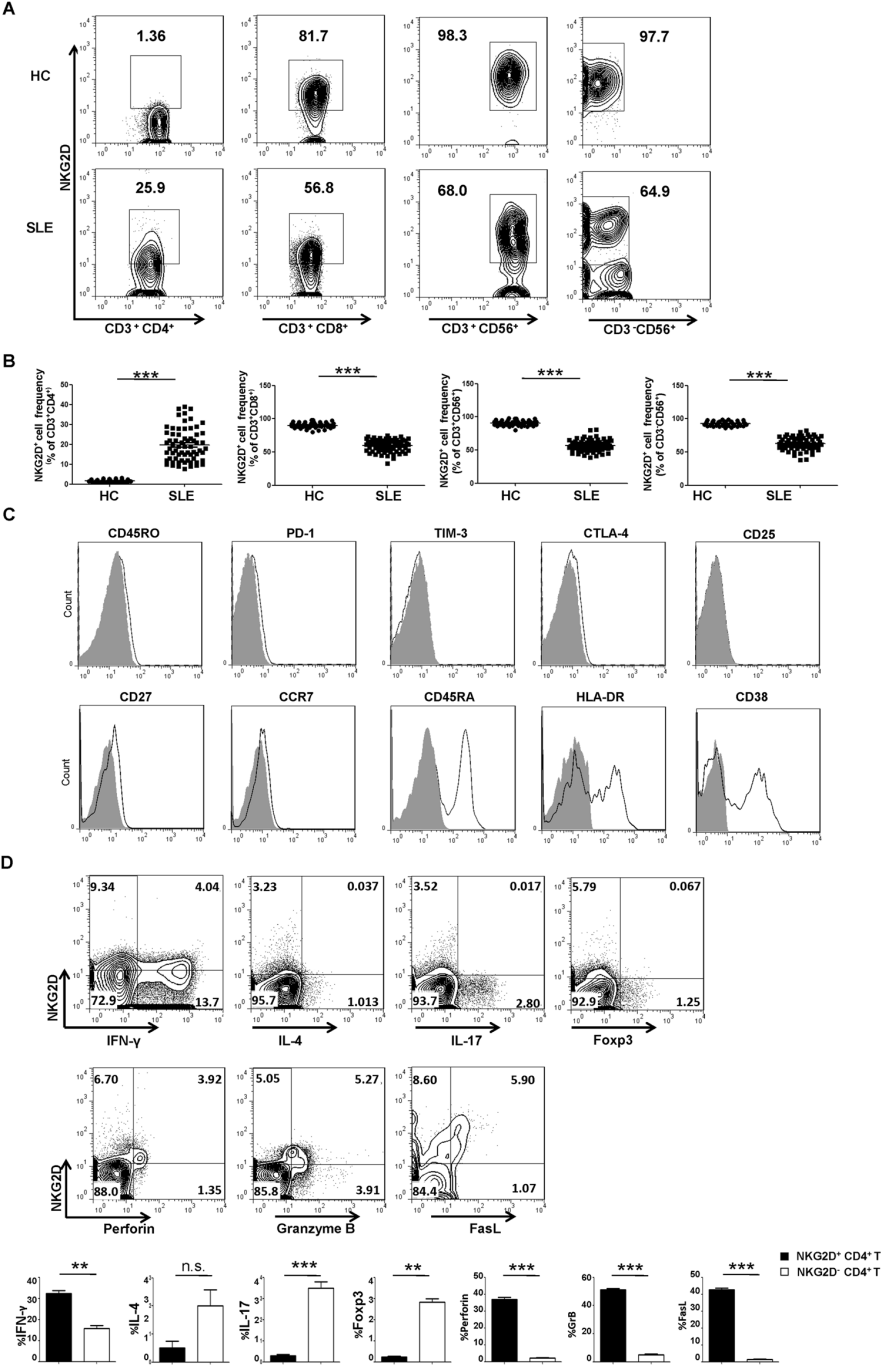
Identification and phenotypic characterization of NKG2D^+^CD4^+^ T cells in SLE. (**A**) FCM assay of NKG2D expression among CD4^+^ T, CD8^+^ T, NK and NKT cells in representative peripheral blood samples from a representative HC and SLE patient. (**B**) Frequency profiles of NKG2D expression on CD4^+^ T, CD8^+^ T, CD3^−^CD56^+^ NK and CD3^+^CD56^+^ NKT cells in the HCs (n = 46) and SLE patients (n = 66). Each symbol represents one individual. (**C**) NKG2D^+^CD4^+^ T cells were gated from a representative peripheral blood sample of an SLE patient and analyzed for expression of the indicated surface markers (black-lined histograms). Gray-filled histograms represent the staining with control Ab. (**D**) FCM assay of the expression of the indicated markers in gated CD4 cells from a representative peripheral blood sample of an SLE patient and statistical analysis of the frequency of the of CD4^+^ T cell subset in PBMCs of SLE patients (n = 30) and HCs (n = 30), respectively. Numbers represent percentages. Each symbol represents one individual; ^**^ *P* < 0.01, ^***^ *P* < 0.001. Horizontal lines with bars show the mean ± SD.

NKG2D, the activating NK receptor, is mainly expressed on cytotoxic cells such as NK, NKT and CD8^+^ T cells, to maintain normal homeostasis, and CD4^+^ T cells express very low levels of this receptor in healthy individuals^32–34^. Recent studies have shown an increased percentage of NKG2D^+^CD4^+^ T cells in some AIDs, such as rheumatoid arthritis, Crohn’s disease and GPA^13–15^, indicating a possible involvement in AID pathogenesis; however, the underlying mechanism remains largely unclear. In this study, SLE patients showed significantly decreased expression of NKG2D on NK (62.58% ± 1.17 vs HC: 92.91% ± 0.50), NKT (56.88% ± 1.08 vs HC: 90.59% ± 0.60) and CD8^+^ T (59.39% ± 1.13 vs HC: 90.02% ± 0.62) cell subsets, and remarkably increased expression on CD4^+^ T cell subsets (19.79% ± 1.01 vs 1.70% ± 0.09) (Fig. 1A and B), suggesting the involvement of NKG2D^+^CD4^+^ T cells in SLE disease progression.

To further define the characteristics of NKG2D^+^CD4^+^ T cells in patients with SLE, we detected the activated surface markers and intracellular cytokine profiles of NKG2D^+^CD4^+^ T cells from SLE patients. These cells did not express CD25, CTLA-4, TIM-3, PD-1, CD45RO, CCR7 or CD27, but they did express CD45RA, HLA-DR and CD38 (Fig. 1C). Of note, these cells showed distinct cytotoxicity and pro-apoptotic features, as approximately 40–50% of these cells expressed perforin, granzyme B and FasL (Fig. 1D), indicating that these cells represent an activated form rather than the naïve state. In addition, these cells did not express the transcription factor Foxp3 or Th2 nor any of the Th17 cell-related cytokines; instead, approximately 30% of the cells secreted IFN-γ, suggesting Th1-like characteristics (Fig. 1D).

### Cytotoxic NKG2D^+^CD4^+^ T cells correlated with decreased frequency of Treg cells in both SLE patients and B6.MRL/lpr mice

**Figure 2:**
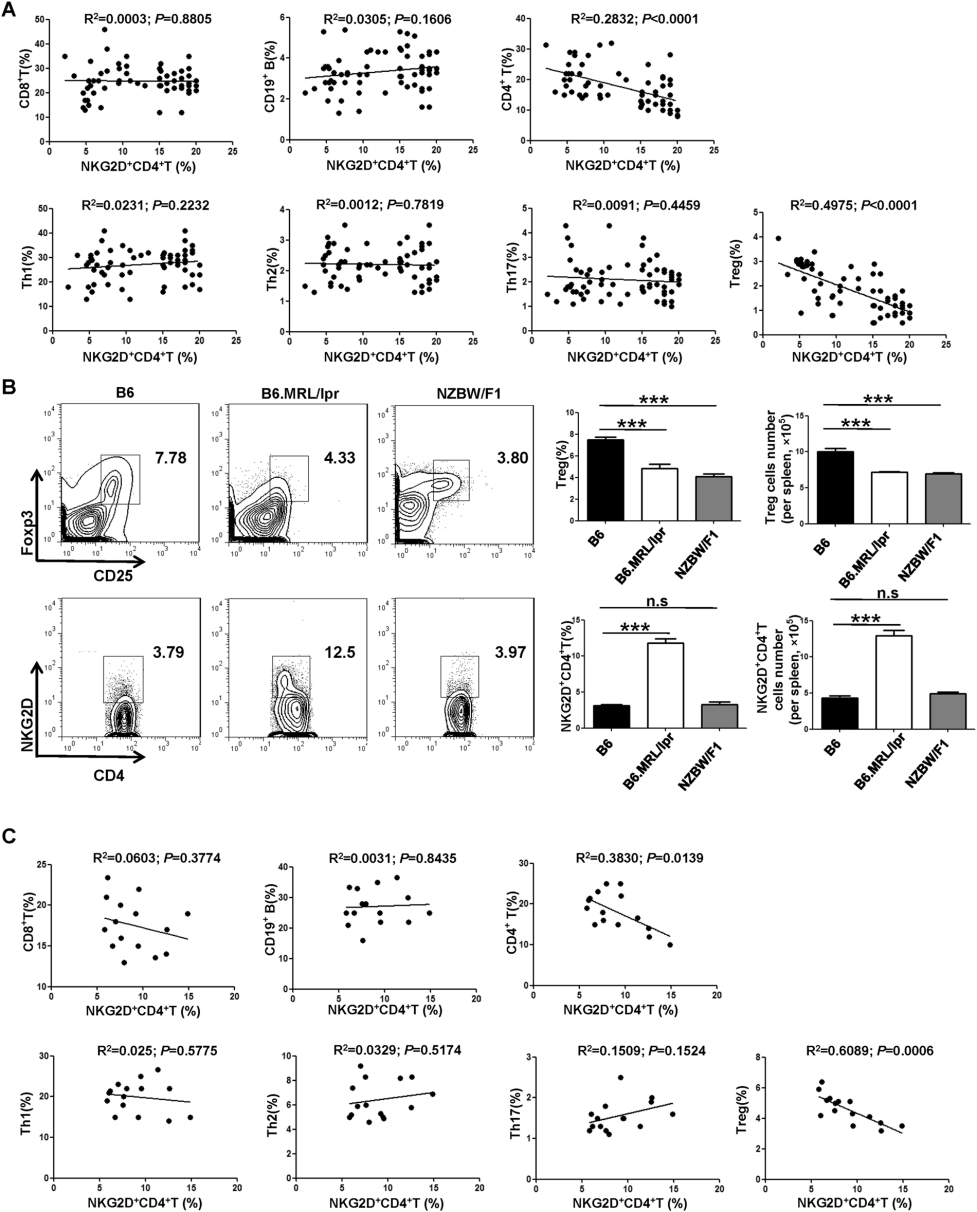
Inverse correlation between the frequencies of NKG2D-expressing CD4^+^ T cells and Treg cells in both SLE patients and MRL/lpr mice. (**A**) Correlation analysis of the frequencies of NKG2D^+^CD4^+^ T cells and T cell subsets and B cells in SLE patients (n = 66). The correlation coefficient and P value are indicated. (**B**) Representative and statistical data of the frequency or cell numbers of Tregs and NKG2D^+^CD4^+^ T cells in splenocytes from B6.MRL/lpr mice, NZBW/F1 mice and C57L/B6 control mice (B6). Data represent three independent experiments with 3–5 mice each. Numbers represent percentages. Horizontal lines with bars show the mean ± SD. ^***^ *P* < 0.001. (**C**) Correlation analysis of the frequencies of NKG2D^+^CD4^+^ T cells and T cell subsets and B cells in B6.MRL/lpr mice. The correlation coefficient and P value are indicated.

A hallmark of human SLE is lymphocytopenia^35^. In line with this observation, we found that the proportions of total T cells (CD3^+^), CD4^+^ T cells (CD3^+^CD4^+^), NK cells (CD3^−^CD56^+^), NKT cells (CD3^+^CD56^+^) and Treg cells (CD4^+^CD25^+^ Foxp3^+^) were dramatically decreased (Supplementary Figure 1), while frequencies of Th1 cells (CD3^+^CD4^+^ IFN-γ^+^), Th2 cells (CD3^+^CD4^+^ IL-4^+^) and Th17 cells (CD3^+^CD4^+^ IL-17^+^) were increased in PBMCs of patients with SLE, as compared with HCs (Supplementary Figure 1B and C). We then analyzed the correlation of the increased NKG2D^+^CD4^+^ T cells subsets with these abnormal proportions of lymphocyte subsets in SLE patients. A strong negative correlation was found between the frequencies of NKG2D^+^CD4^+^ T cells and CD4^+^ T cells (R^2^ = 0.2832, *P* < 0.0001) in the PBMCs of SLE patients, but no correlation was found for the frequency of CD8^+^ T cells or CD19^+^ B cells (Fig. 2A). Among the CD4^+^ T subsets, the frequency of NKG2D^+^CD4^+^ T cells was found to be negatively correlated with the frequency of Treg cells (R^2^ = 0.4975, *P* < 0.0001), but not with the frequency of Th1, Th2 or Th17 cells (Fig. 2A), suggesting that the aberrant expansion of NKG2D^+^CD4^+^ T cells correlates with a decrease in Treg cells and with CD4^+^ T cell frequencies in SLE patients. As NKG2D is also expressed on CD4^−^ cells, including the CD8^+^ T, NK and NKT cells, we then analyzed the correlation between these cells with Treg cells and the results showed no obvious correlation between the frequency of NKG2D^+^CD8^+^ T cells, NKG2D^+^ NK cells or NKG2D^+^ NKT cells with Treg cells in SLE patients (Supplementary Figure 2).

B6.MRL/lpr and NZBW/F1 mice are universal lupus animal models^5, 6^ and both showed decreased frequency of Treg cells or of absolute cell numbers in spleen; however, we found that the B6.MRL/lpr mice but not the NZBW/F1 mice showed an increased frequency of NKG2D^+^CD4^+^ T cells in our experimental period (Fig. 2B). Therefore, we adopted the B6.MRL/lpr lupus mouse model for this study, since they could mimic the clinical features of SLE patients with respect to NKG2D^+^CD4^+^ T cells. We then analyzed the correlation of the NKG2D^+^CD4^+^ T cells with the various types of lymphocytes in the B6.MRL/lpr mice. Similar results were obtained as in SLE patients, which showed NKG2D^+^CD4^+^ T cells frequency is inversely correlated to the frequency of CD4^+^ T cells (R^2^ = 0.3830, *P* = 0.0139) and Treg cells (R^2^ = 0.6089, *P* = 0.0006), respectively (Fig. 2C). This finding further confirmed the close correlation between NKG2D^+^CD4^+^ T cells and Treg cells in SLE.

### NKG2D^+^CD4^+^ T cells from SLE patients efficiently killed NKG2DL Treg cells *in vitro*

**Figure 3:**
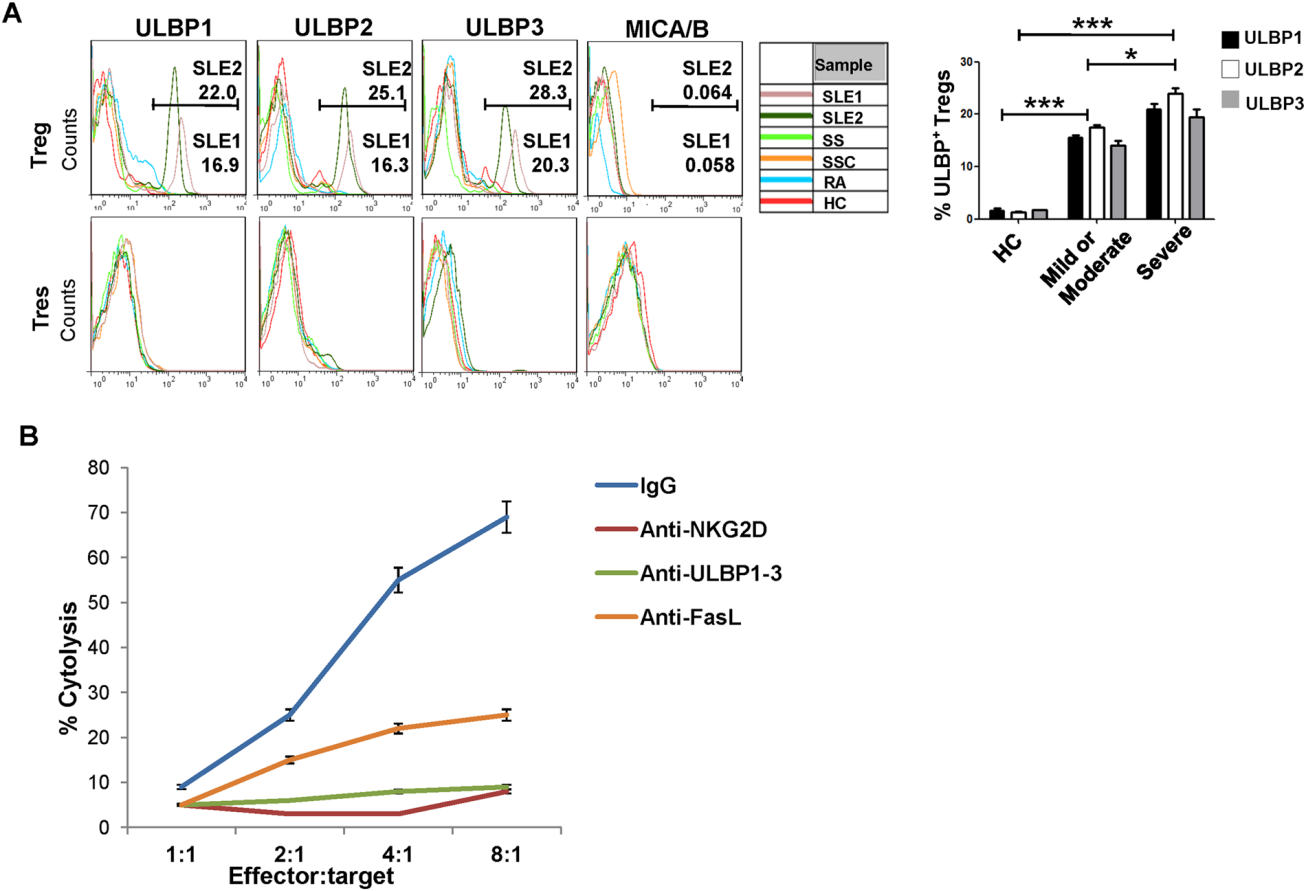
NKG2D^+^ CD4^+^ T cells killed Treg cells had upregulated NKG2DLs *in vitro*. (**A**) Induction of ULBP expression on Treg cells by the indicated serum samples. Treg cells were isolated from PBMCs of healthy controls (HCs) and stimulated *in vitro* by the addition of 10% serum from SLE (n = 10), Sjogren syndrome (SS) (n = 5), systemic scleroderma (SSc) (n = 8), rheumatoid arthritis (RA) (n = 8) patients, or HC (n = 9), respectively, for 18 h, followed by FCM assay. CD4^+^CD25^−^ T responders (Tres) served as controls. The histograms (left) show original data from one representative experiment. The SLE1 and SLE2 samples are representative serum from patients with mild/moderate (n = 4) and severe (n = 6) SLE, according to SLEDAI index. Bar graphs (right) present the statistical results obtained from three independent experiments with sorted healthy Treg cells stimulated with the indicated serum. ^*^ *P* < 0.05, ^***^ *P* < 0.001. (**B**) Freshly isolated Treg cells from HC were stimulated with the serum of SLE patients, labeled with ^51^Cr and irradiated (25 Gy), co-cultured for 6–8 h with NKG2D^+^CD4^+^ T cells from SLE patients, and subjected to cytotoxicity assay, and assessed for the ^51^Cr-release at the indicated effector:target ratios; the experiment was performed with or without pre-incubation with the indicated neutralizing antibodies. Data represent three independent experiments with Treg cells from HC.

Based on the aberrant expansion of NKG2D^+^CD4^+^ T cells and its negative correlation with Treg cells, we purified Treg cells from HCs and induced the NKG2DL ULBP1-3 expression by stimulation with SLE serum for 18 h *in vitro* (Fig. 3A). The NKG2D^+^CD4^+^ T cells from the SLE patients efficiently killed the NKG2DL^+^ Treg cells (Fig. 3B), accounting for the negative expression of NKG2DLs on CD3^+^CD4^+^ T cells from SLE patients (Supplementary Figure 3). Furthermore, this cytotoxicity was NKG2D/NKG2DL-dependent since the addition of blocking antibodies against NKG2D or the NKG2DLs, ULPB1-3, significantly downregulated the cytotoxicity (Fig. 3B). The underlying effector mechanism, at least partially, involved the Fas/FasL apoptosis pathway, since addition of neutralizing antibodies against FasL markedly reduced this effect (Fig. 3B). In addition, monocytes that express NKG2DLs were isolated from SLE patients for use as the positive control of target cells. Results showed that these monocytes could be killed by NKG2D^+^CD4^+^ T cells (Supplementary Figure 4), suggesting that the cytotoxicity of NKG2D^+^CD4^+^ T cells involved various targets in SLE.

Notably, we found that the induction of the NKG2DLs ULBP1-3 expression was specific for SLE serum, since serum from patients with Sjogren syndrome, systemic scleroderma or rheumatoid arthritis did not induce the expression of NKG2DLs on Treg cells (Fig. 3A). Moreover, when we categorized the SLE serum according to mild/moderate or severe disease status (according to the SLEDAI index), we found higher expression of ULBPs on Tregs induced by SLE serum from severe patients, as compared to that from mild/moderate patients (Fig. 3A). However, SLE serum did not induce the expression of any NKG2DLs on CD4^+^CD25^-^ T responder (Tres) cells (Fig. 3A).

### Adoptive transferred NKG2DL^+^ Treg cells were killed by NKG2D^+^CD4^+^ T cells in B6.MRL/lpr mice

**Figure 4:**
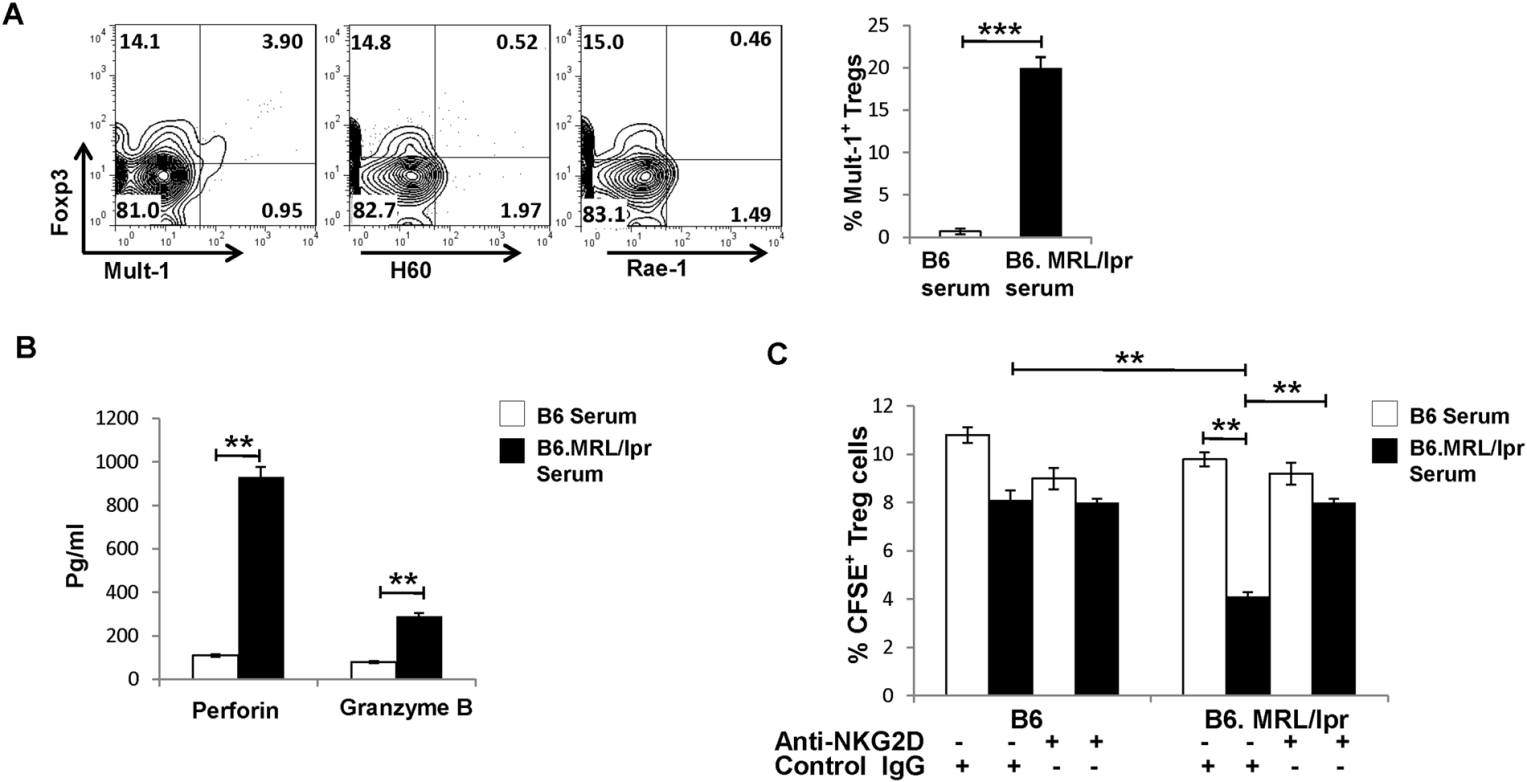
NKG2D^+^CD4^+^ T cells killed NKG2DLs-expressing Treg cells in B6.MRL/lpr lupus mice. (**A**) Induction of NKG2DLs expression on mouse Treg cells by lupus mouse serum. Treg cells isolated from the spleens of C57BL/6 (B6) mice were stimulated with B6 or B6.MRL/lpr lupus mouse serum and then assessed by FCM. Data (left) shown are representative of results from lupus mouse serum. Bar graphs (right) are the statistical results obtained from three independent experiments conducted with 3–5 mice. Numbers represent percentages. (**B**) Treg cells isolated from the spleens of B6 mice were stimulated with serum from B6 control mice or from B6.MRL/lpr lupus mice and then co-cultured for 6–8 h with NKG2D^+^CD4^+^ T cells from B6.MRL/lpr lupus mice. Supernatants were assessed by ELISA. Bar graphs present the statistical results obtained from three independent experiments with Treg cells and NKG2D^+^CD4^+^ T cells. (**C**) CFSE-labeled Treg cells from B6 mice were stimulated *in vitro* with B6 serum or MRL/lpr mouse serum and transferred to B6 mice and B6.MRL/lpr mice, with or without indicated antibodies pre-treatment, respectively, and the frequency of exogenous Treg cells was examined in gated CD4^+^ T cells by FCM. Data represent three independent experiments with 3–5 mice. Horizontal lines with vertical bar borders show the mean ± SD. ^**^ *P* < 0.01, ^***^ *P* < 0.001.

Similar to the findings of SLE patients (Supplementary Figure 3), both B6.MRL/lpr and B6 control mice showed undetectable expression of NKG2DLs (Mult-1, the counterpart of human ULBPs; H60; Rae-1) on the CD3^+^CD4^+^ T cells (Supplementary Figure 5). To further investigate whether the NKG2D^+^CD4^+^ T cells exert similar cytotoxic effects on NKG2DL^+^ Treg cells *in vitro* and *in vivo*, we stimulated Treg cells isolated from B6 control mice with B6.MRL/lpr mice serum and found that the NKG2DL Mult-1, but not H60 or Rae-1, was significantly upregulated (Fig. 4A). ELISA assay showed marked increase of perforin and granzyme B derived from the NKG2D^+^CD4^+^ T cells co-cultured with lupus serum that stimulated Treg cells, indicating that NKG2D^+^CD4^+^ T cells could kill target cells by secreting cytotoxic mediators *in vitro* (Fig. 4B). Moreover, after adoptive transfer tothe B6.MRL/lpr mice, the NKG2DL^+^ Treg cells were efficiently killed by NKG2D^+^CD4^+^ T cells *in vivo* (Fig. 4C), and pre-treatment with anti-NKG2D mAb in MRL/lpr mice showed an obvious restoration of the exogenous Treg cells frequency (Fig. 4C). All these results indicated that the cytotoxicity of NKG2D^+^CD4^+^ T cells on NKG2DL^+^ Treg cells contributed to the remarkable decrease in Treg cells frequency in lupus.

### IFN-α/IFNα receptor (IFNAR) pathway was mainly responsible for the induction of NKG2DLs expression on Treg cells

**Figure 5:**
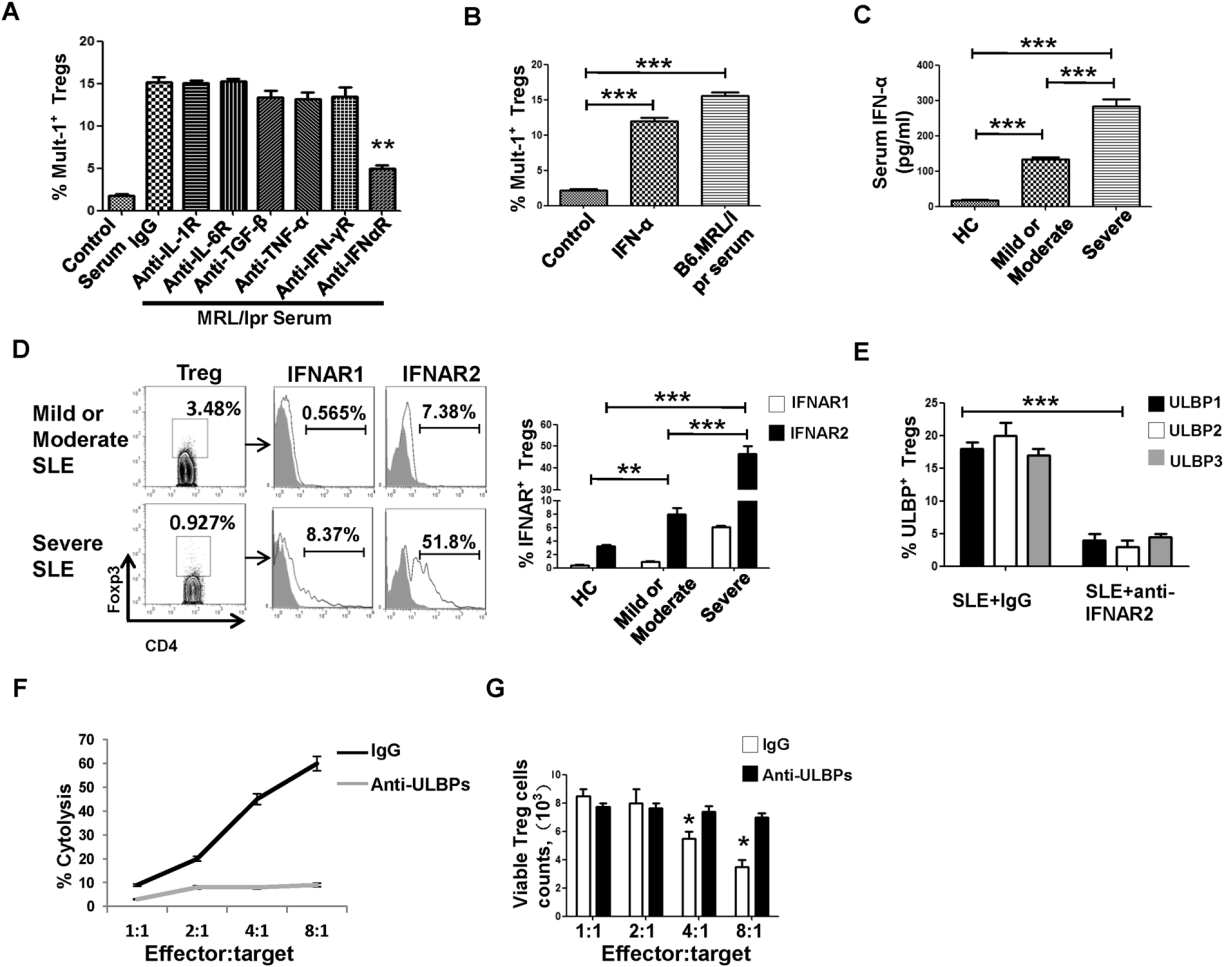
IFN-α/IFNAR signaling induced expression of NKG2DLs on Treg cells. (**A**) Frequencies of Mult-1 expression on Treg cells stimulated by B6.MRL/lpr mouse serum, in the presence or absence of indicated antibodies, were analyzed. (**B**) Frequencies of Mult-1 expression on Treg cells stimulated in the presence or absence recombinant murine IFN-α (1000 U/mL) were analyzed. B6.MRL/lpr mouse serum were used as positive controls. (**C**) The levels of serum IFN-α were measured by ELISA for 30 HCs, 36 mild/moderate SLE patients and 26 severe SLE patients. (**D**) FCM assay results for the frequency of Treg cells from representative mild/moderate SLE patients and severe SLE patients. The gated Treg cells were further investigated for IFNAR1/R2 expression. The representative FCM assay (left) of expression of IFNAR on Treg cells are shown as indicated (black solid line). Filled gray histograms represent the staining with control Abs. Bar graphs (right) present the statistical results of the IFNAR^+^ Treg cells ratio for Treg cells isolated from 30 HCs, 36 mild/moderate SLE patients and 26 severe SLE patients. (**E**) Frequencies of expression of ULBPs on Treg cells stimulated by serum from SLE patients with different disease activity (n = 25), in the presence or absence of anti-IFNAR2 mAb (5 μg/mL), were analyzed. (**F**) Cytolysis percentage and (**G**) Treg cells count in the co-culture system with NKG2D^+^CD4^+^ T cells and IFN-α stimulated Treg cells at different ratios of effector:target cells, with or without the neutralized antibodies, measured by the ^51^Cr-release assay and apoptosis assay, respectively. All values represent the mean ± SD of three independent experiments. ^**^ *P* < 0.01, ^***^ *P* < 0.001.

The above results indicated that SLE serum induced expression of NKG2DLs on Treg cells. To further investigate the cytokine types that may be involved in the NKG2DLs induction, we firstly measured the levels of IFN-α, IFN-γ, IL-6, IL-1β and TNF-α in B6.MRL/lpr mice serum by ELISA. It was found that, although all these cytokines showed elevated levels in lupus serum (Supplementary Figure 6A), only anti-IFNAR Abs were able to obviously downregulate the induction of NKG2DL Mult-1 expression (Fig. 5A), which was consistent with the finding of high IFNAR expression on Treg cells in MRL/lpr mice (Supplementary Figure 6B). Accordingly, recombinant murine IFN-α remarkably induced Mult-1 expression on Treg cells isolated from B6.MRL/lpr mice, which was comparable with the induction effects by B6.MRL/lpr serum (Fig. 5B). All these data suggested that, IFN-α was the major cytokine responsible for the induction of expression of NKG2DLs on the Treg cells in B6.MRL/lpr mice.

To further investigate whether the IFN-α/IFNAR pathway also plays a critical role in the induction of NKG2DLs ULBPs on human Treg cells, we measured IFN-α in SLE patients’ serum and IFNAR expression on Treg cells, it was found that IFN-α levels (Fig. 5C; *P* < 0.0001) and IFNAR1/R2 expression in SLE patients were remarkably increased compared with HCs (Fig. 5D; IFNAR1: *P* = 0.0090 and IFNAR2: *P* < 0.0001). Moreover, severe SLE patients showed higher serum levels of IFN-α (Fig. 5C, *P* < 0.0001) and IFNAR1/R2 expression than the mild/moderate SLE patients (Fig. 5D; IFNAR1: *P* < 0.0001 and IFNAR2: *P* < 0.0001). Blocking IFN-α/IFNAR signaling by anti-IFNAR mAb remarkably downregulated the ULBPs induction on Treg cells (Fig. 5E). All these data indicated that IFN-α/IFNAR signaling is responsible, at least partially, for the induction of NKG2DLs on Treg cells in patients with SLE.

Furthermore, we performed co-culture assay to demonstrate that IFN-α is directly involved in the mechanism underlying the increased ULBPs expression on Treg cells, and thus leading to the reduction of Treg cells in SLE. Treg cells from healthy controls were stimulated with IFN-α and the viable Treg cells were sorted and co-cultured with NKG2D^+^CD4^+^ T cells, with or without anti-ULBPs mAbs. Results showed that NKG2D^+^CD4^+^ T cells could efficiently kill these Treg cells and that blocking the NKG2D-ULBPs interaction by anti-ULBPs mAbs significantly inhibited the cytolysis effect on Treg cells (Fig. 5F), which was further supported by the absolute counts of viable Treg cells in the co-culture system (Fig. 5G). It should be noted that we also found that IFN-α could induce apoptosis of Treg cells, that the frequency of apoptotic Treg cells was markedly increased, as compared to that of the PBS control (Supplementary Figure 7A), and that the viable Treg cell frequency was significantly decreased by SLE serum, as compared to HC serum (Supplementary Figure 7B). Collectively, these data indicated that the IFN-α/IFNAR pathway is a primary mediator of the induction of NKG2DLs on Treg cells and, thereby, the reduction of Treg cells in patients with SLE. In contrast to these findings with Tregs, CD45RO^+^CD4^+^ memory T cells could only slightly induce the ULBPs expression by IFN-α (Supplementary Figure 8).

### Blockade of IFN-α/IFNAR or NKG2D restored Treg cells frequency and alleviated lupus disease in B6.MRL/lpr mice

**Figure 6:**
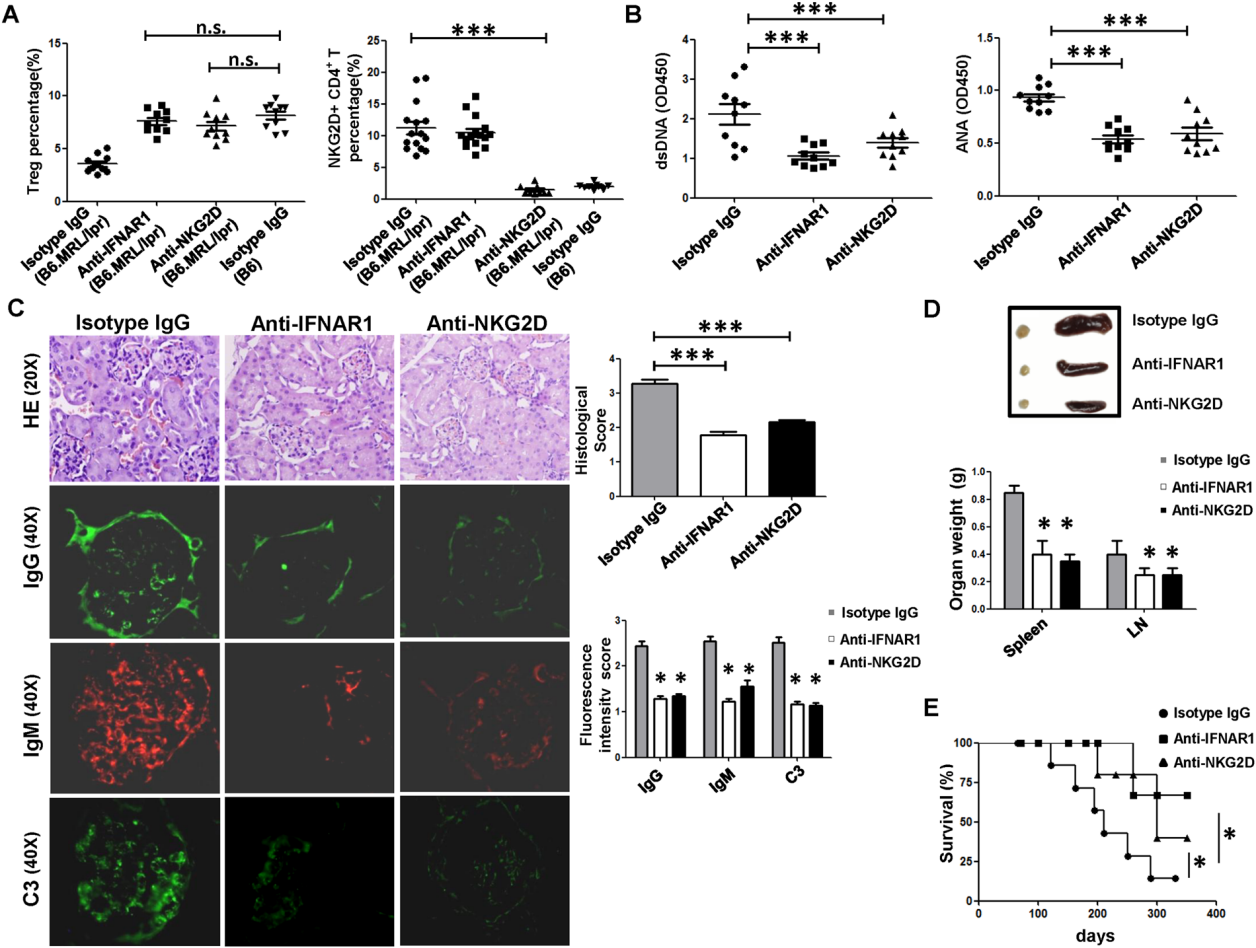
Anti-IFNAR mAb and anti-NKG2D mAb treatment ameliorated lupus disease in B6.MRL/lpr mice. (**A**) Comparison of the frequencies of Treg cells and NKG2D^+^CD4^+^ T cells in B6.MRL/lpr mice after 5 wk of IFNAR and NKG2D antibody treatments, as well as in healthy B6 control mice. (**B**) Serum level of autoantibodies (anti-dsDNA and ANA) after IFNAR and NKG2D antibody treatments in B6.MRL/lpr mice. Data in A and B are presented as mean ± SD. (**C**) Kidney histology (left) showing reduced glomerular hypercellularity and immune deposits (IgG, IgM and C3) after IFNAR and NKG2D antibody treatments in B6.MRL/lpr mice. Histological score and fluorescence intensity score (right) were assessed. Results are representative of three experiments with 5–6 mice per group. (**D**) Weights of spleen and lymph nodes (LN) after IFNAR1 and NKG2D antibody treatments in B6.MRL/lpr mice. Data are presented as mean ± SD. (**E**) Survival analysis for the IFNAR and NKG2D antibody treatments in B6.MRL/lpr mice, as compared with PBS treatment. Each symbol represents one individual, and data are representative of three independent experiments. ^*^ *P* < 0.05, ^**^ *P* < 0.01, ^***^ *P* < 0.001.

The above results indicated that the reduction of Treg cells in SLE was involved with the increased NKG2DLs expression induced by IFN-α, so we treated B6.MRL/lpr mice with neutralizing anti-IFNAR mAb or anti-NKG2D mAb. The results showed that the frequency of the Treg cells was restored to a level comparable to that in C57BL/6 control mice after 5 successive weeks of anti-IFNAR mAb treatment (7.62% vs 8.14%, *P* = 0.32) or anti-NKG2D mAb (7.14% vs 8.14%, *P* = 0.1) (Fig. 6A). Furthermore, most disease parameters showed significant reduction. Compared to isotype IgG treatment, anti-IFNAR mAb or anti-NKG2D mAb treatment of B6.MRL/lpr mice led to a significant reduction in serum levels of the antinuclear antibodies (ANA) autoantibodies (anti-IFNAR mAb: *P* < 0.0001 and anti-NKG2D mAb: *P* = 0.0083; Fig. 6B) and ds-DNA (anti-IFNAR mAb: *P* < 0.0001 and anti-NKG2D mAb: *P* = 0.0006; Fig. 6B), an obvious improvement in glomerulonephritis (GN) with decreased glomerular hypercellularity and mesangial cell proliferation, a remarkable down-regulation in the kidney deposits (IgG, IgM and C3) (Fig. 6C), a marked reduction in splenomegaly and lymphadenophthy (Fig. 6D), and a significant increase in the survival rate (Fig. 6E).

## Discussion

SLE patients show a significantly decreased Treg cell frequency, which might be responsible for the severe immune homeostasis disorder that defines this AID^2–6^. Nevertheless, the mechanism underlying the decrease in Treg cells number remains unclear. In this study, SLE patient samples and the B6.MRL/lpr lupus mouse model were both found to have an association between the significantly decreased Treg cell frequency and marked expansion in the NKG2D^+^CD4^+^ T cell population. Moreover, the NKG2D^+^CD4^+^ T cells were found to express cytotoxic and proapoptotic factors such as perforin, granzyme B and FasL, which efficiently killed Treg cells that were induced by IFN-α to express NKG2DLs *in vitro*. Furthermore, adoptively transferred NKG2DL^+^ Treg cells were also found to be killed in the B6.MRL/lpr lupus mice in a NKG2D-NKG2DL dependent manner. Anti-IFNAR or anti-NKG2D treatment of B6.MRL/lpr mice remarkably restored Treg cell numbers and ameliorated the lupus disease, confirming the substantial contribution of NKG2D^+^CD4^+^ T cells to the decrease in Treg cells observed in SLE patients.

In contrast to the significantly decreased NKG2D expression on CD8^+^ T, NK and NKT cells, we observed a marked increase in NKG2D^+^CD4^+^ T cells in SLE patients, in both the present study and in our previous work^16^. It has been demonstrated in several other studies that sTNF-α and sIL-15, which are abundant in inflamed tissue or peripheral blood, can induce NKG2D expression on CD4^+^ T cells^13, 16, 17^. Moreover, NKG2D^+^CD4^+^ T cells have been demonstrated to exhibit cytotoxic features in Crohn’s disease, being capable of killing target C1R cells (a human B-cell lymphoblastoid line that lacks surface HLA A and B antigens) that had been transfected with MICA *in vitro* ^14^. NKG2D^+^CD4^+^ T cells are also able to kill vascular endothelial cells in GPA disease^17^. In the present study, we demonstrated that NKG2D^+^CD4^+^ T cells from SLE patients secreted perforin and granzyme B, as well as the Th1-cytokine IFN-γ, mirroring the features of the NKG2D^+^CD4^+^ T cells in Crohn’s disease and GPA^14, 17^. Furthermore, the NKG2D^+^CD4^+^ T cells were able kill SLE serum-induced NKG2DL^+^ Treg cells in a NKG2D-dependent manner. This is the first study to demonstrate that the abnormally increased NKG2D^+^CD4^+^ T cells of SLE can kill NKG2DL^+^ Tregs, and the findings contrast the previous report of chimeric NKG2D-transduced CD8^+^ T cells killing NKG2DL^+^ Treg cells in a murine model of ovarian cancer^36^.

Although it has been demonstrated that NK cells could lyse expanded Treg cells stimulated by tuberculosis bacteria (T.B.) in an NKG2D-dependent manner^28^, those findings were obtained from experiments using the *in vitro* T.B. stimulation assay. As such, that study was able to confirm that cytokine-activated NK cells could kill stimulated Tregs, but it did not reflect the *in vivo* state of NK cells in SLE patients. Actually, in SLE patients, the frequencies of NKT and NK cells are decreased significantly, as shown in this study and in the previously reported literature^37^; although the exact mechanisms underlying these decreases remain unknown. In addition, the expression of NKG2D is significantly reduced on CD8^+^ T, NKT and NK cells in SLE, as demonstrated by our and previous studies^37, 38^. Therefore, in the SLE context, the CD8^+^ T, NKT and NK cells have defects that affect their abilities to kill the target cells in an NKG2D-dependent manner. This detrimental effect has been demonstrated by studies of other AIDs, infectious diseases and tumors^32, 39–41^. Moreover, we found that the frequency of NKG2D^+^CD4^−^ cells, including CD8^+^ T, NK or NKT cell types, did not correlate with that of Treg cells in SLE patients. In contrast, the frequency NKG2D^+^CD4^+^ T cells and expression of NKG2D on them were both significantly increased, showing strong cytolytic function towards Treg cells in the *in vitro* and *in vivo* assays. Taken together, considering the dysfunction or decreased cell count of NKG2D^+^CD4^−^ cells, our results imply that the killing of Tregs is NKG2D^+^CD4^+^ T cell-specific in the SLE context.

It is important to consider that Dai *et al*.^23^ reported that NKG2D^+^CD4^+^ T cells in juvenile-onset SLE produced IL-10 and TGF-β, thus having regulatory function. These inconsistencies with our results could be explained by the clinical subject selection; we chose treatment-naive adult patients, while Dai’s group chose treatment-experienced (prednisone) juvenile patients, and as such could not exclude the effect of prednisone on NKG2D^+^CD4^+^ T cells. In addition, in the present study, we found that NKG2D^+^CD4^+^ T cells from SLE patients could kill monocytes that express MICA/B and ULBP1-3, further indicating the cytolytic capacity of NKG2D^+^CD4^+^ T cells towards NKG2DLs-expressing target cells in SLE patients.

Spontaneous B6.MRL/lpr and NZBW/F1 mouse models have been used extensively in the studies of immunological mechanisms and therapeutic targets over the years^5, 6^. Each of these models exhibit high levels of serum ANAs and anti-dsDNA antibodies and develop GN. The B6.MRL/lpr mice have a loss-of-function lymphoproliferation mutation (i.e. *lpr*) within the gene encoding Fas, a cell-surface protein that mediates apoptosis^42^. Defects in Fas signaling allow for increased double-negative CD4^−^CD8^−^CD3^+^B220^+^ T cell population, thereby leading to lymphadenopathy and splenomegaly^43^. Therefore, the B6.MRL/lpr mice present with dysregulation of T cells and develop AID. The NZBW/F1 lupus mice carry the lupus-susceptibility genes (e.g. *Sle1* and *Sle3*) that are responsible for expansion of autoreactive B cells and autoantibody production^44–46^. However, in the current study, we observed significantly increased frequency and cell number of NKG2D^+^CD4^+^ T cells in B6.MRL/lpr mice, but not in NZBW/F1 mice. This observation might be due to the completely different genetic backgrounds of the mice or to the different time of lupus development in these two models, both of which could affect NKG2D^+^ cell numbers; however, the exact mechanisms underlying the distinctive increase of NKG2D^+^CD4^+^ T cells in B6.MRL/lpr mice but not in NZBW/F1 mice remain unclear and need to be clarified in future studies.

ULBPs and MICA/B are ligands for NKG2D in humans that are expressed at low levels by many tissues^19^, and expression is upregulated primarily on epithelial cells, mononuclear phagocytes^20^ and dendritic cells^22^ under stress by infection or malignant transformation^20, 21^. Spada *et al*.^24^ reported increased expression of MICA in kidneys of patients with lupus nephritis and of Rae-1γ and Mult-1 expression in the MRL/lpr lupus mouse model; however, they detected the expression of these NKG2DLs by immunohistochemistry assay and thus could only locate the NKG2DL expression in the glomerular cells in general, but not in any specific cell subset. Dai *et al*.^23^ demonstrated the increased serum level of sMICA in SLE patients but did not determine the source cell of the sMICA. In the present study, we confirmed that there was no detectable expression of NKG2DLs on T cell subsets from SLE patients; however, we did observe that induction by SLE serum or IFN-α could upregulate ULBP1-3 expression, but not MICA/B expression, on Treg cells. Therefore, the increased sMICA level in SLE patients^23^ might derive from other immune cells than the Treg cells.

Type I IFN signaling is a key pathogenic pathway in SLE^47^. It has been reported that treatment of the NZB mouse model with IFN-α or the IFN-α inducer polyinosinic:polycytidylic acid (poly I:C) to address AID resulted in significantly decreased Treg cell frequency^48^. Similarly, in our *in vitro* stimulation assay, we observed induction of Treg cell apoptosis by IFN-α or SLE serum which included high level of IFN-α. However, we were able to exclude effects of apoptosis in the cytotoxicity assay because we performed cell co-culture and adoptive transfer experiments after sorting only viable cells labeled with Annexin V and a vital dye. Therefore, our results clearly demonstrated that the viable Treg cells induced to express ULBP or Mult-1 by IFN-α were lysed by NKG2D^+^CD4^+^ T cells rather than by IFN-α-induced apoptosis.

Type I IFNs have been implicated as important players in SLE pathogenesis and exposure to anti-IFN-α mAb results in improvement of SLE disease activity^49, 50^. Yet, several studies have reported the reverse function of IFN-α in lupus mouse models^30, 51, 52^, indicating that there are no IFN-α signature profiles for the splenic mononuclear cells of MRL/lpr mice^51^, that IFNAR antibody has no effect^30^ and that IFNAR deletion results in even more disease in MRL/lpr mice^52^. It is important to note that all these studies used the MRL/lpr lupus mouse model, which was generated from a series of crosses with strains C57BL/6J (0.3%), C3H/HeDi (12.1%), AKR/J (12.6%) and LG/J (75%)^43^. It should be also be noted that, in our study, we observed substantial protective roles for IFNAR antibody treatment in B6.MRL/lpr mice with C57BL/6 background.

Braun *et al*.^53^ also reported that sustained injection of poly I:C, a potent inducer of IFN-I, into B6.MRL/lpr mice resulted in dramatic aggravation of the renal disease, higher titers of autoantibodies and accumulation of activated lymphocytes. Similarly, Ramani *et al*.^54^ reported that poly I:C could induce IFN-I-dependent crescentic GN in B6.MRL/lpr mice, indicating that IFN-I signaling contributed to the worsened SLE disease of these mice. Moreover, introducing a null mutation for the IFN-I receptor gene into the B6.MRL/lpr background mice resulted in dramatically decreased immune complex deposition in the kidney and in reduced lymphadenopathy^53^. Thus, the difference in potential for IFN-α function in lupus might be dependent upon the different mouse models used in each study, suggesting that explanation of the related results is founded in the genetic background of the involved mice.

Compared with the well-developed IFN-α antibody therapy in SLE^49, 50^, we found that NKG2D antibody treatment has similar effects in B6.MRL/lpr mice, consistent with previous findings in mouse models of non-obese diabetes, collagen-induced arthritis and colitis^31, 55, 56^. A recent report on clinical trials of NKG2D antibody against Crohn’s disease revealed a significant reduction of disease activity after several weeks of treatment^57^. The underlying mechanism might involve blockade of the NKG2D-NKG2DL interaction and avoidance of activation of the NKG2D^+^ T, NK or NKT cells^57^. Nevertheless, NKG2D expression on NK, NKT and CD8^+^ T cells was markedly decreased in SLE patients, while the CD4^+^ T cells showed increased NKG2D expression. The mechanism underlying the amelioration of SLE disease after NKG2D antibody treatment might involve blockade of the interaction between NKG2D^+^CD4^+^ T cells and Treg cells, thereby causing restoration of the Treg frequency as observed in treated mice. Given that NKG2DLs are extensively expressed on non-T cells, such as monocytes and glomeruli, in SLE or lupus nephritis^16, 24^, NKG2D antibody therapy would also be expected to block the mutual activation of these cells with NKG2D^+^CD4^+^ T cells. Thus, subsequent studies should investigate the precise mechanism of this significant amelioration in SLE achieved by NKG2D antibody therapy and should identify more specific targets for clinical therapy for SLE.

In conclusion, our findings reveal unique contributions of NKG2D^+^CD4^+^ T cells and their killing effect on ULBP^+^ (Mult-1^+^) Treg cells in the pathogenesis of SLE and highlight the NKG2D-NKG2DL interaction in SLE. These results improve our understanding of SLE clinical phenomena and will guide future studies of targeted therapies in SLE patients.

## Electronic supplementary material

Supplementary Figures and Tables without change marks
