# Supplementary material for: NKG2D+CD4+ T Cells Kill Regulatory T Cells in a NKG2D-NKG2D Ligand- Dependent Manner in Systemic Lupus Erythematosus
Source: Sci Rep. 2017 Apr 28;7:1288. doi: 10.1038/s41598-017-01379-y (PMC5430709; doi:10.1038/s41598-017-01379-y)
Supplement: Supplementary file 1 — Supplementary Figures and Tables without change marks [file 41598_2017_1379_MOESM1_ESM.doc]

**Supplementary data**

**NKG2D^+^CD4^+^ T Cells Kill Regulatory T Cells in a NKG2D-NKG2D Ligand-Dependent Manner in Systemic Lupus Erythematosus**

Di Yang^1^, Zhiqiang Tian^1^, Mengjie Zhang^4^, Weibing Yang^2^, Jun Tang^3,*^, Yuzhang Wu^1,*^, Bing Ni^4,1,*^

^1^Institute of Immunology, PLA, Third Military Medical University, Chongqing 400038, PR China

^2^Department of Dermatology, 181^st^ Hospital of PLA, Guilin 541002, PR China

^3^Department of Dermatology, 105^th^ Hospital of PLA, Hefei 230001, PR China

^4^Department of Pathophysiology and High Altitude Pathology/Key Laboratory of High Altitude Environment Medicine (Third Military Medical University), Ministry of Education/Key Laboratory of High Altitude Medicine, College of High Altitude Military Medicine, Third Military Medical University, Chongqing 400038, PR China

**Supplementary Tables**

Sppl. Table 1. Characteristics of the patients with system lupus erythematosus*

| Characteristic | Patients with mild or moderate disease (n=40) | Patients with severe disease (n=26) | Healthy controls  (n=46) |
| --- | --- | --- | --- |
| Age, mean ± SD years | 45 ± 11 | 50 ± 13 | 48 ± 12 |
| Sex, n male/female | 10/30 | 6/20 | 20/26 |
| CD4^+^NKG2D^+^/CD4, mean ± SD | 19.9 ± 7.7 | 18.9 ± 8.8 | 1.67 ± 0.59 |
| Treg/ CD4, mean ± SD | 2.3 ± 0.5 | 1.1 ± 0.3 | 5.7 ± 0.1 |
| SLEDAI score, mean ± SD | 8 ± 3 | 18 ± 4 | - |

*Disease activity was classified as mild, moderate, or severe according to the Systemic Lupus Erythematosus Disease Activity Index (SLEDAI) score (scores of 5–9 were considered mild, scores of 10–14 were considered moderate, and scores of >14 were considered severe).

**Supplementary Figures**


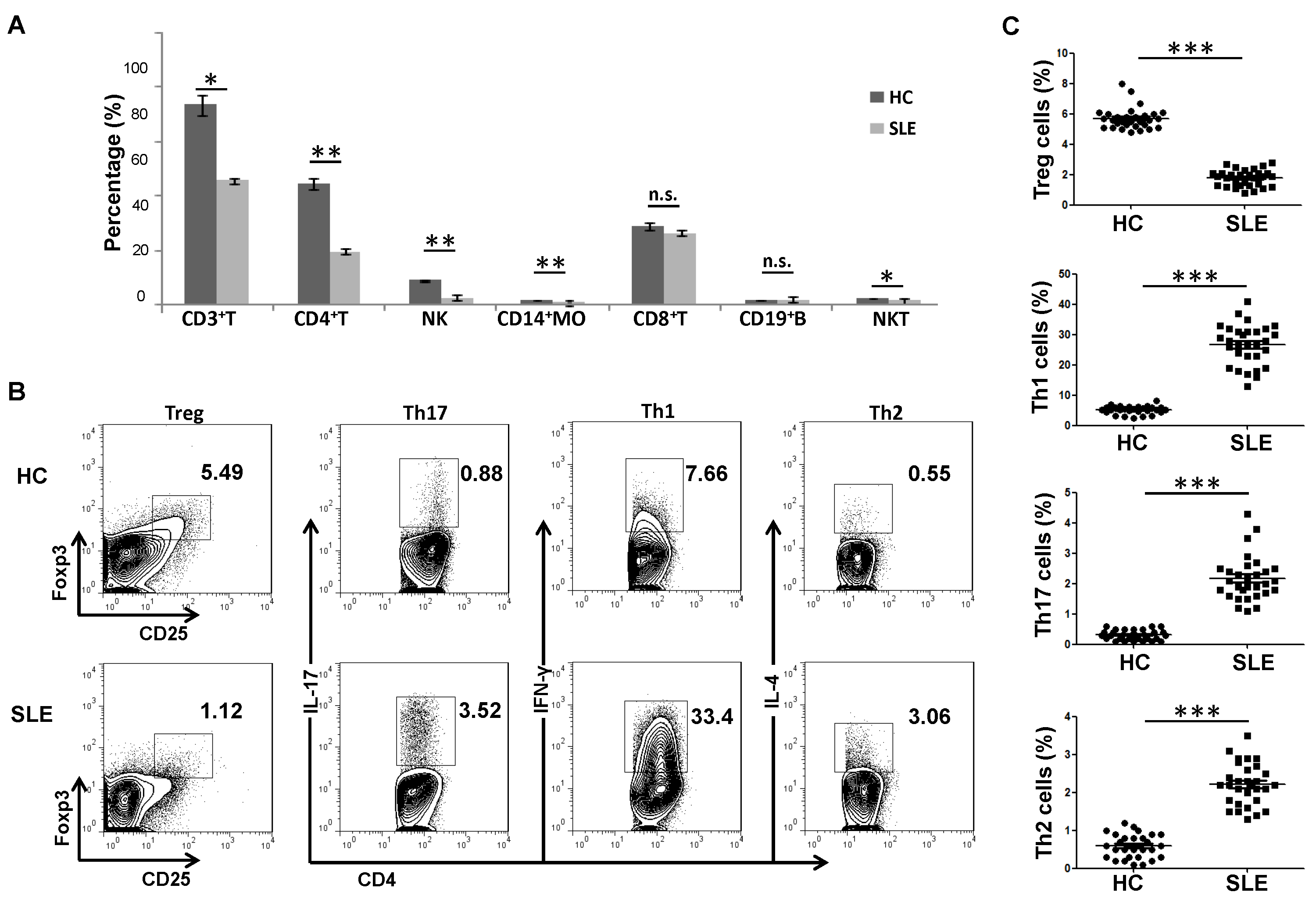


**Figure S1. Aberrant proportions of leukocyte subsets in PBMCs from SLE patients.**

(**A**) Summary of the frequency of lymphocyte and monocyte subsets in the PBMCs of 66 SLE patients (shaded bars) and 46 healthy controls (HCs) (solid bars). Horizontal lines with bars show the mean ± SD. (**B**) Representative frequency of different types of Th cells in gated CD4^+^ T cells in a peripheral blood sample from SLE and HC. Numbers represent percentages. (**C**) Statistical analysis of the frequency of the of CD4^+^ T cell subset in PBMCs of SLE patients (n=30) and HC (n=30), respectively. Each symbol represents one individual; ^*^ *P*<0.05, ^**^ *P*<0.01, ^***^ *P*<0.001.


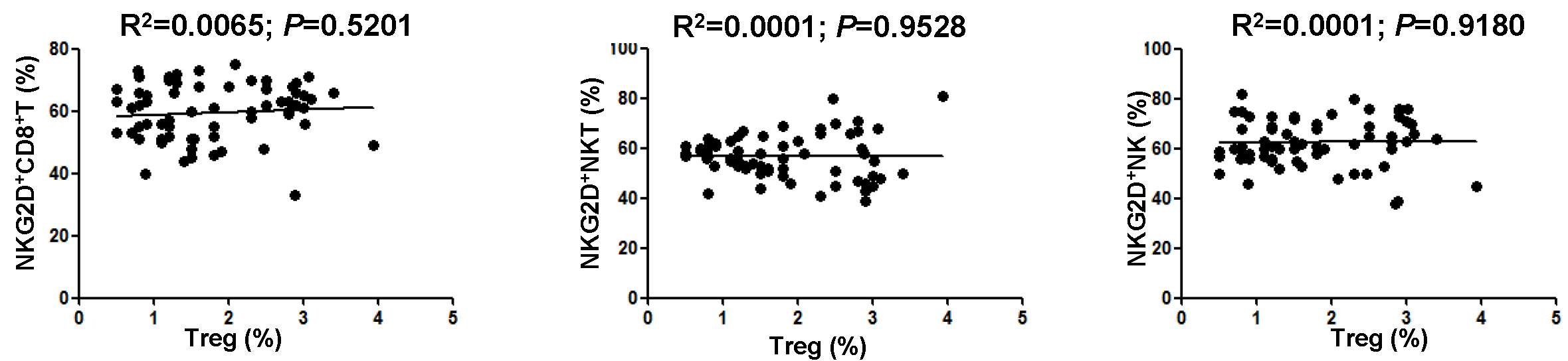


**Figure S2. Correlation** **analysis of the frequencies of NKG2D-expressing CD4^-^ T cells and Treg cells in patients with SLE.**

SLE patients (n=66). The correlation coefficient and *P* value are indicated.


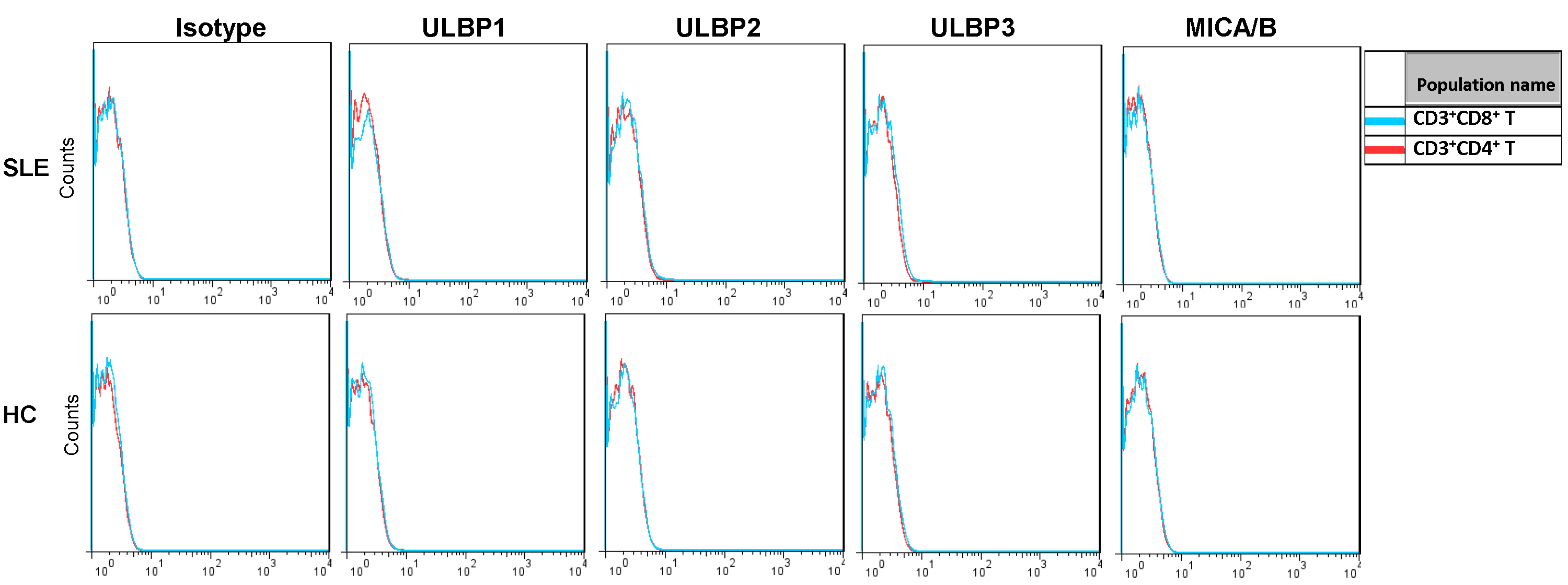


**Figure S3. Expression of NKG2D ligands on freshly isolated peripheral blood mononuclear cells (PBMCs) of SLE patients and healthy controls.**

Mononuclear cells were isolated from peripheral blood of SLE patients and healthy controls (HCs) and FCM assay of expression of NKG2D ligands on gated CD3^+^CD4^+^ T cells and CD3^+^CD8^+^ T cells are shown as indicated. The histograms are representative of duplicate experiments in cells from 30 independent SLE patients and 30 healthy donors.


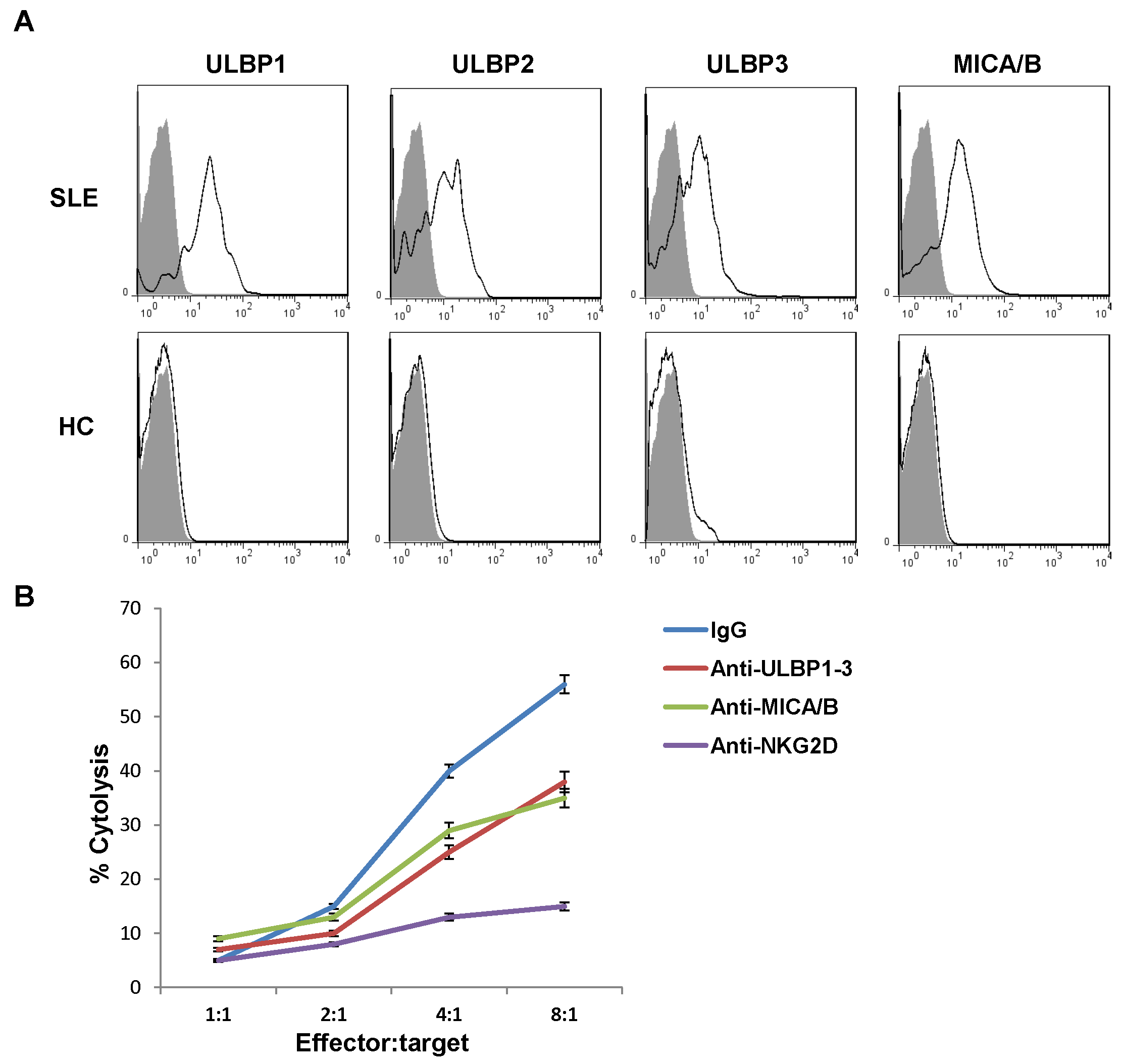


**Figure S4. NKG2D^+^CD4^+^ T cells killed NKG2D ligands-expressing monocytes *in vitro.***

(**A**) Expression of NKG2D ligands on fresh isolated monocytes of healthy controls (HCs) (n=15) and patients with SLE (n=25). Mononuclear cells were isolated from peripheral blood of SLE patients and HCs for FCM assay. The representative results of expression of NKG2D ligands on gated CD14^+^ monocytes are shown (black solid line). Filled gray histograms represent control staining with IgG. (**B**) Cytolysis of monocytes by NKG2D^+^CD4^+^ T cells. Freshly isolated monocytes from SLE patients were labeled with ^51^Cr and irradiated (25Gy), co-cultured for 6-8h with NKG2D^+^CD4^+^ T cells from SLE patients, and subjected to cytotoxicity assay and to measurement of the^51^Cr released at the indicated effector:target ratios, with or without pre-incubation with the indicated neutralizing antibodies. Data represent three independent experiments with monocytes and NKG2D^+^CD4^+^ T cells.

**
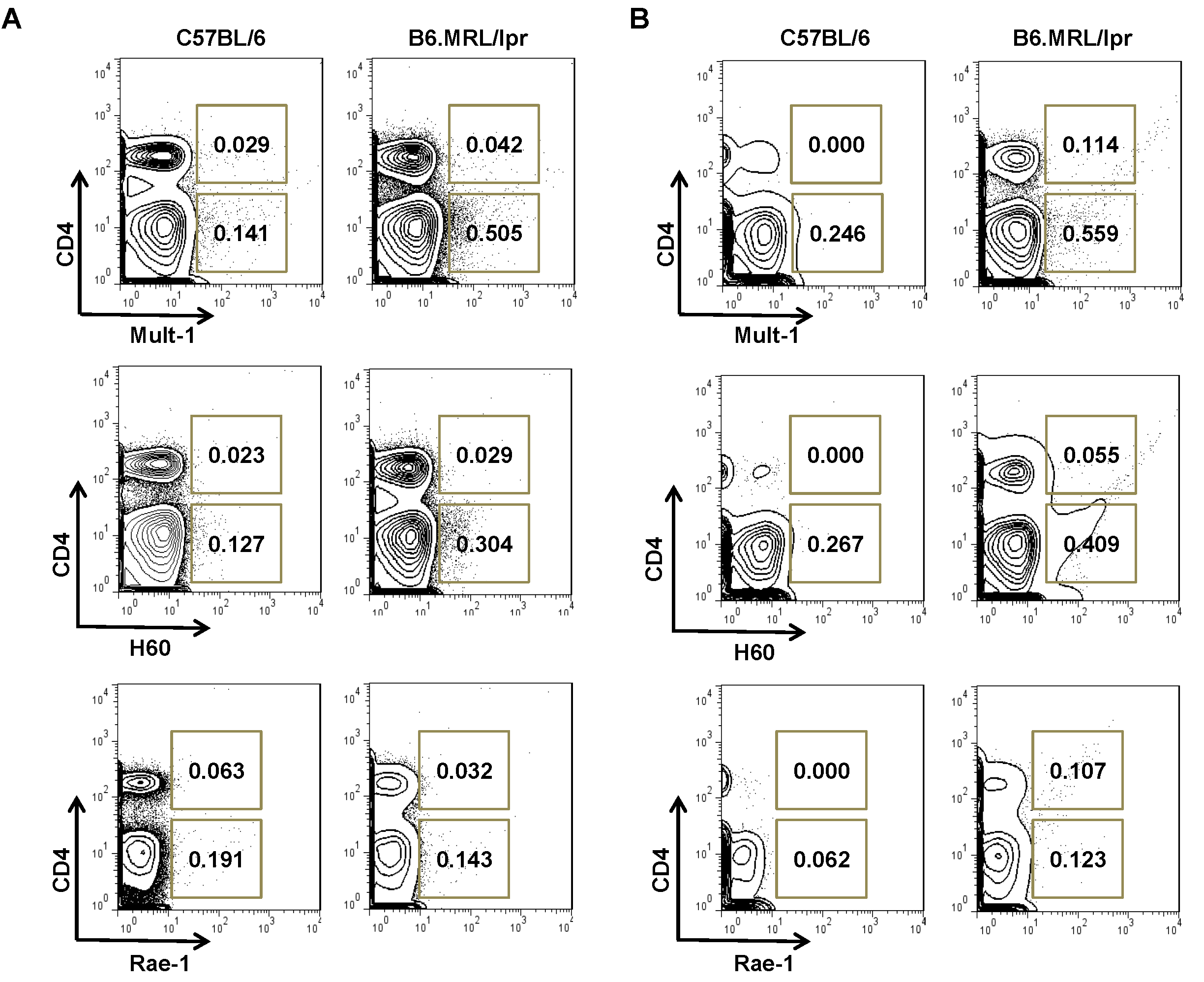
**

**Figure S5. NKG2D ligands expression profiles in lupus mice.**

(**A**) and (**B**) Expression of NKG2D ligands on CD4^+^ or non-CD4^+^ cells among mononuclear cells isolated from spleen (**A**) and kidney (**B**) of lupus mice and C57BL/6 (B6) controls, respectively. Representative data from C57BL/6 mice and B6.MRL/lpr mice are shown and the percentages are indicated.


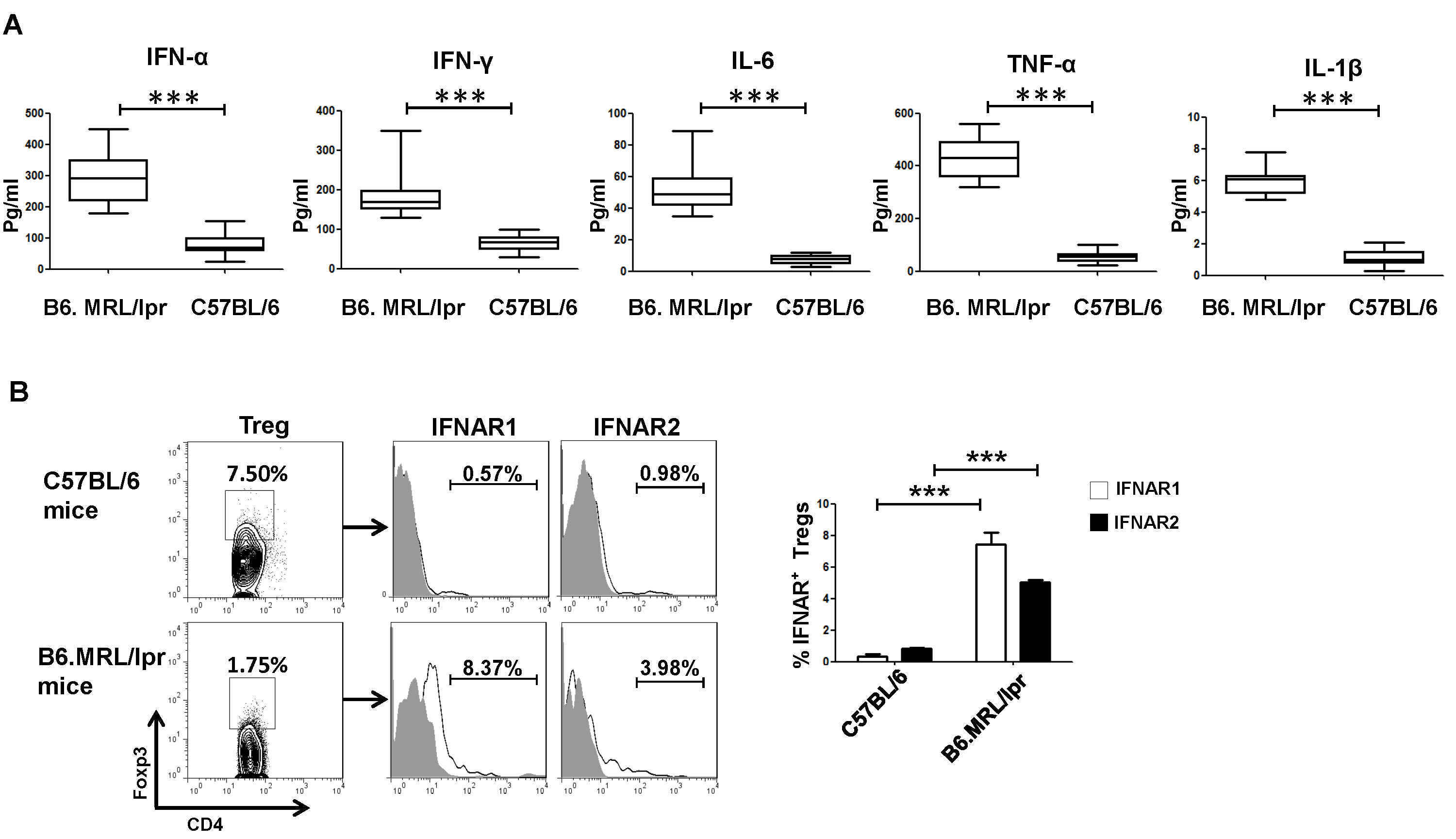


**Figure S6. Elevated serum level of pro-inflammatory cytokines and IFNAR expression on Tregs in lupus mice.**

**(A)** Elevated serum level of pro-inflammatory cytokines (as indicated) in and MRL/lpr mice, as assessed by ELISA. **(B)** Frequency of Treg cells from B6.MRL/lpr mice and B6 mice, as assessed by FCM. The gated Treg cells were further investigated for IFNAR1/R2 expression. The representative FCM assay (left) of IFNAR expression on Treg cells is shown (black solid line). Filled gray histograms represent staining with control IgG. Bar graphs (right) are statistical results of IFNAR expression from three independent experiments with 5–6 mice per group. All values are the mean ± SD of three independent experiments. ^***^ *P*<0.001.


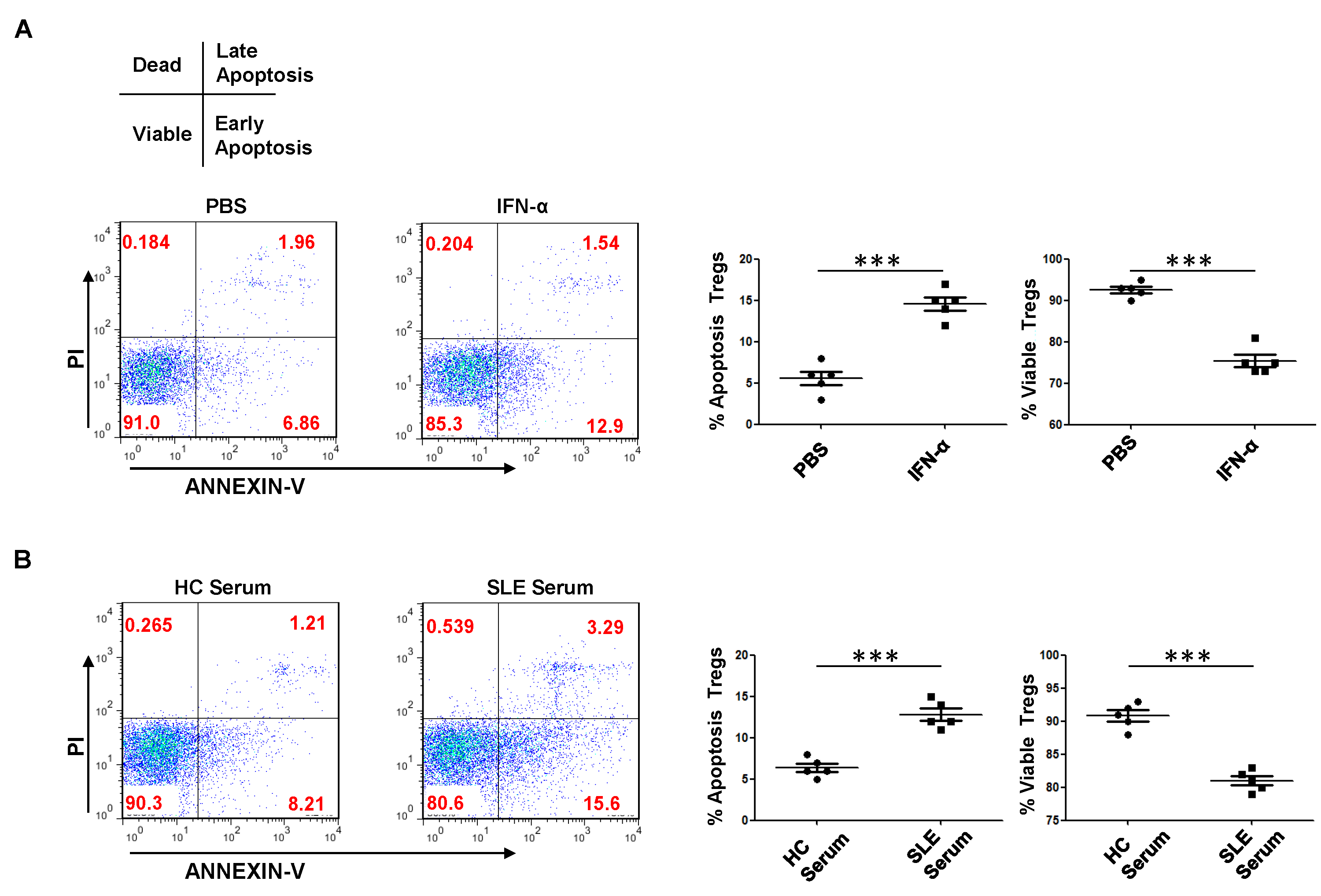


**Figure S7. Induction of Treg cell apoptosis by IFN-α or SLE serum.**

Treg cells sorted from healthy controls (HCs) were stimulated by IFN-α (**A**) or serum from patients with SLE (**B**). The apoptosis effect was assessed by FCM using propidium iodide and Annexin V staining. Scatter plots are the representative data of three independent experiments. All values are mean ± SD of three independent experiments. ^***^ *P*<0.001.


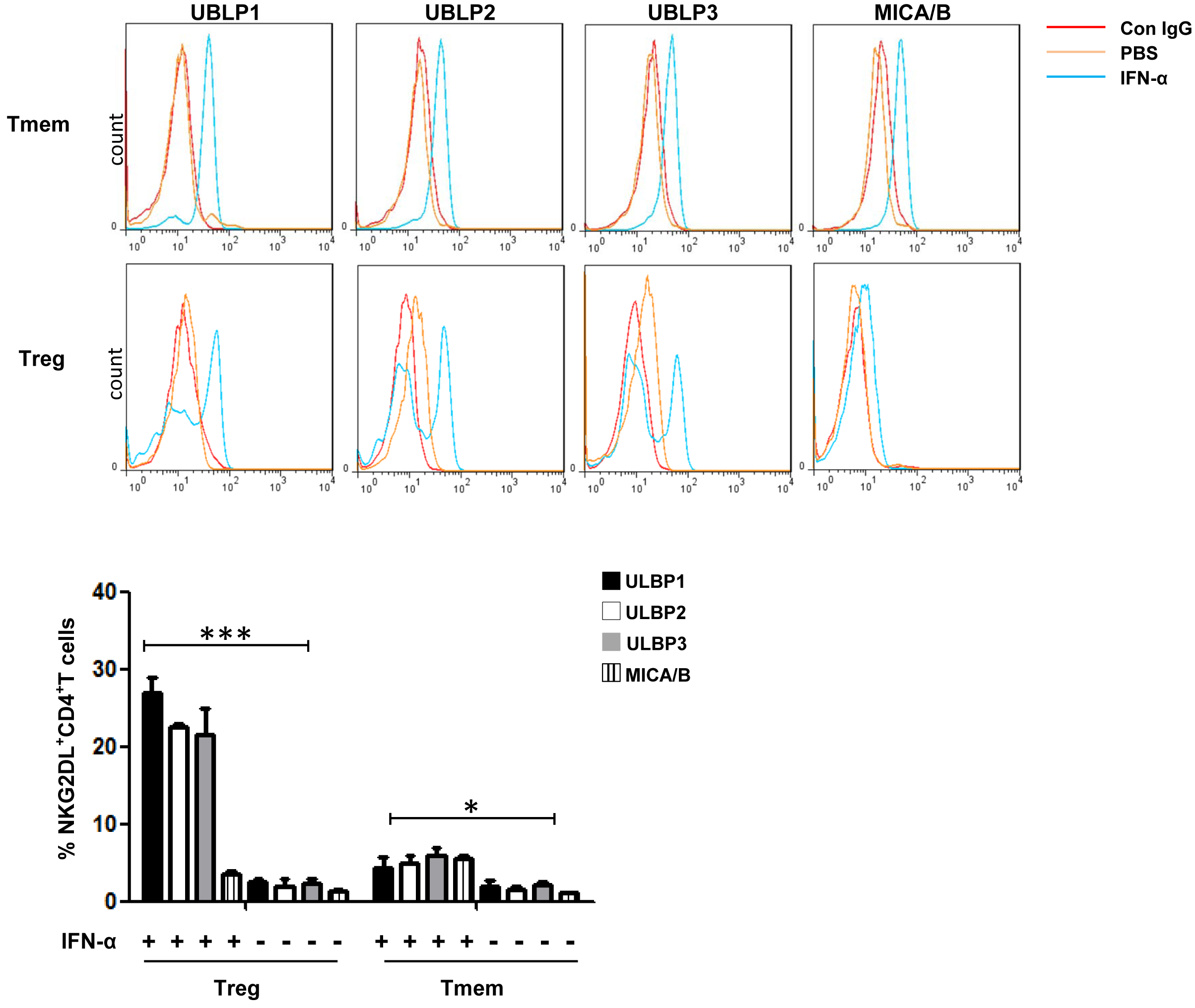


**Figure S8. Induction of NKG2D ligands expression on Treg and CD4^+^CD45RO^+^ memory T cells by IFN-α.**

Treg cells and CD45RO^+^CD4^+^ memory T cells were freshly isolated from healthy controls, induced by IFN-α, and the expression of NKG2D ligands on these cells was detected by FCM assay. Histograms are the representative data of NKG2D ligands expression on Treg cells and CD45RO^+^CD4^+^ memory T cells (upper panel). The frequency of NKG2DL^+^CD4^+^ T cells were statistically assessed (lower panel). All values are the mean ± SD of three independent experiments. ^*^ *P*<0.05, ^***^ *P*<0.001.
